# Impact of vagus nerve cross-sectional area on electrocardiogram parameters in community-dwelling older adults: The YAHABA study

**DOI:** 10.1371/journal.pone.0353473

**Published:** 2026-07-10

**Authors:** Nao Onodera, Kazumasa Oura, Hiroshi Akasaka, Naoki Ishizuka, Tohru Fujiwara, Ryo Itabashi, Tetsuya Maeda

**Affiliations:** 1 Division of Neurology and Gerontology, Department of Internal Medicine, School of Medicine, Iwate Medical University, Yahaba, Shiwa, Iwate, Japan; 2 Central Clinical Analysis Division, Iwate Medical University Hospital, Yahaba, Shiwa, Iwate, Japan; 3 Department of Laboratory Medicine and Infection Disease, School of Medicine, Iwate Medical University, Yahaba, Shiwa, Iwate, Japan; University of Missouri, UNITED STATES OF AMERICA

## Abstract

**Objectives:**

The aim of this study was to investigate the association between vagus nerve cross-sectional area (VN-CSA) using carotid ultrasonography and electrocardiogram (ECG) parameters in community-dwelling older adults, thereby clarifying the influence of VN-CSA on cardiac electrical activity.

**Methods:**

A cross-sectional study examining the association between VN-CSA and ECG parameters. We prospectively enrolled participants aged ≥65 years who underwent both 12-lead ECG and carotid ultrasonography from the 2023 survey of a community-based cohort study (YAHABA study). The VN-CSA was measured offline at the thyroid level using carotid ultrasonography with a 3–8 MHz linear probe. Participants were divided into tertiles of each side; right tertile (RT)1 (<1.0 mm), RT2 (1.0–1.1 mm), and RT3 (>1.1 mm^2^). and left tertile (LT)1 (<0.9 mm^2^), LT2 (0.9–1.0 mm^2^), and LT3 (>1.0 mm^2^). ECG parameters were compared among the three groups on each of the left and right sides. Furthermore, linear regression analysis was performed to examine the association between left and right VN-CSA and ECG parameters.

**Results:**

Finally, 183 participants enrolled in this study. In the analysis of covariance in multiple comparisons among tertiles of each side, RT1 had a higher heart rate (p = 0.021), a shorter R–R interval (p = 0.037), and a higher P-wave amplitude (p = 0.008) than RT2, while no significant differences were observed between RT1 and RT3. No significant associations were observed for the left VN-CSA. In the linear regression analyses, significant associations were observed between the left VN-CSA and QRS duration (p = 0.038), while there was no significant association between the right VN-CSA and ECG parameters.

**Conclusion:**

The size of the vagus nerves had different effects on cardiac electrical activity in the left and right sides. The right VN-CSA may be associated with the electrical activity of the sinus node and atria, while the left VN-CSA may be associated with the electrical activity of the ventricles.

## Introduction

The prevalence of arrhythmias increases with age, as demonstrated by the Cardiovascular Health Study, which evaluated individuals aged ≥65 years in four regions of the United States and observed a strong association between supraventricular arrhythmias and aging [1]. Periodic health examination data similarly indicate that the prevalence of atrial fibrillation (AF) increases with age [2].

The development of arrhythmias is influenced by structural cardiac changes, lifestyle-related diseases, excessive alcohol intake, smoking, and autonomic nervous system activity. For instance, analyzing heart rate variability (HRV) immediately before paroxysmal AF onset reveals two distinct patterns: (1) increased low-frequency components with decreased high-frequency components, and (2) decreased low-frequency components with increased high-frequency components, suggesting that abrupt changes in autonomic balance trigger AF [3]. Moreover, a significant decrease in high-frequency components precedes the onset of ventricular tachycardia, suggesting that reduced parasympathetic function contributes to ventricular tachycardia [4].

The vagus nerve is responsible for parasympathetic function. Animal studies using vagal stimulation have indicated that right-sided stimulation primarily decreases heart rate, whereas left-sided stimulation induces atrioventricular block [5–8]. Furthermore, another study showed that in patients with heart failure, heart rate decreased significantly after vagus nerve stimulation compared with before stimulation on both the right and left sides, but the reduction was more pronounced on the right side [9]. However, these findings were derived from studies on vagus nerve stimulation; therefore, whether the vagus nerve cross-sectional area (VN-CSA) itself correlates with cardiac electrical activity remains unclear.

Recently, ultrasonographic studies have explored the association between the VN-CSA and autonomic function. Most of these studies have evaluated neurodegenerative diseases or neuropathies, including Parkinson's disease [10–12], amyotrophic lateral sclerosis [13,14], Guillain-Barré syndrome [15], and diabetes mellitus [16–18], and it was reported that the VN-CSA may decrease or increase depending on the disease type, and may correlate with disease duration [13,17]. Although these reports suggest that long-term autonomic dysfunction may be associated with morphological changes in the vagus nerve, these findings are not yet entirely consistent. In addition, it was reported that VN-CSA may be influenced by anatomical and environmental factors [19–21], and studies of healthy individuals have reported normative data based on background factors such as age and body build [22,23]. One report noted a correlation between the left VN-CSA and the parasympathetic parameter HRV in healthy participants, suggesting laterality in the VN-CSA the association with influence on cardiac autonomic function [24]. However, there is limited evidence regarding the association between VN-CSA and physiological parameters in healthy individuals, and the matter remains open to debate.

The aim of this study was to comprehensively investigate the association between the VN-CSA evaluated by carotid ultrasonography and electrocardiogram (ECG) parameters (heart rate, P-wave indices, and various intervals/durations) in community-dwelling older adults, thereby clarifying the influence of VN-CSA on cardiac electrical activity.

## Materials and methods

### Study participants

We enrolled participants from the Yahaba Active Aging and Healthy Brain (YAHABA) study, a prospective cohort study established in 2016 focusing on the development of neurological diseases among individuals aged ≥65 years living in Yahaba, Iwate, Japan. The study enrolled 962 participants in the baseline analysis conducted from 2016 to 2018. Of these, 200 were excluded from the follow-up study owing to death, dropout, or lack of examination. Among the 762 participants who participated in the follow-up study conducted from 2021 to 2023, both carotid echocardiography and 12-lead ECG were performed for the 202 participants who were followed up from January 10, 2023 to March 1, 2023. The exclusion criteria were a history of pacemaker implantation and AF detected by ECG. The study protocol complied with the ethical guidelines of the 2013 Declaration of Helsinki and was approved by the Institutional Ethics Committee of Iwate Medical University (approval no. HG2020−017). Written informed consent was obtained from all participants prior to study enrollment.

### Ultrasonography imaging of the VN-CSA

**Fig 1:**
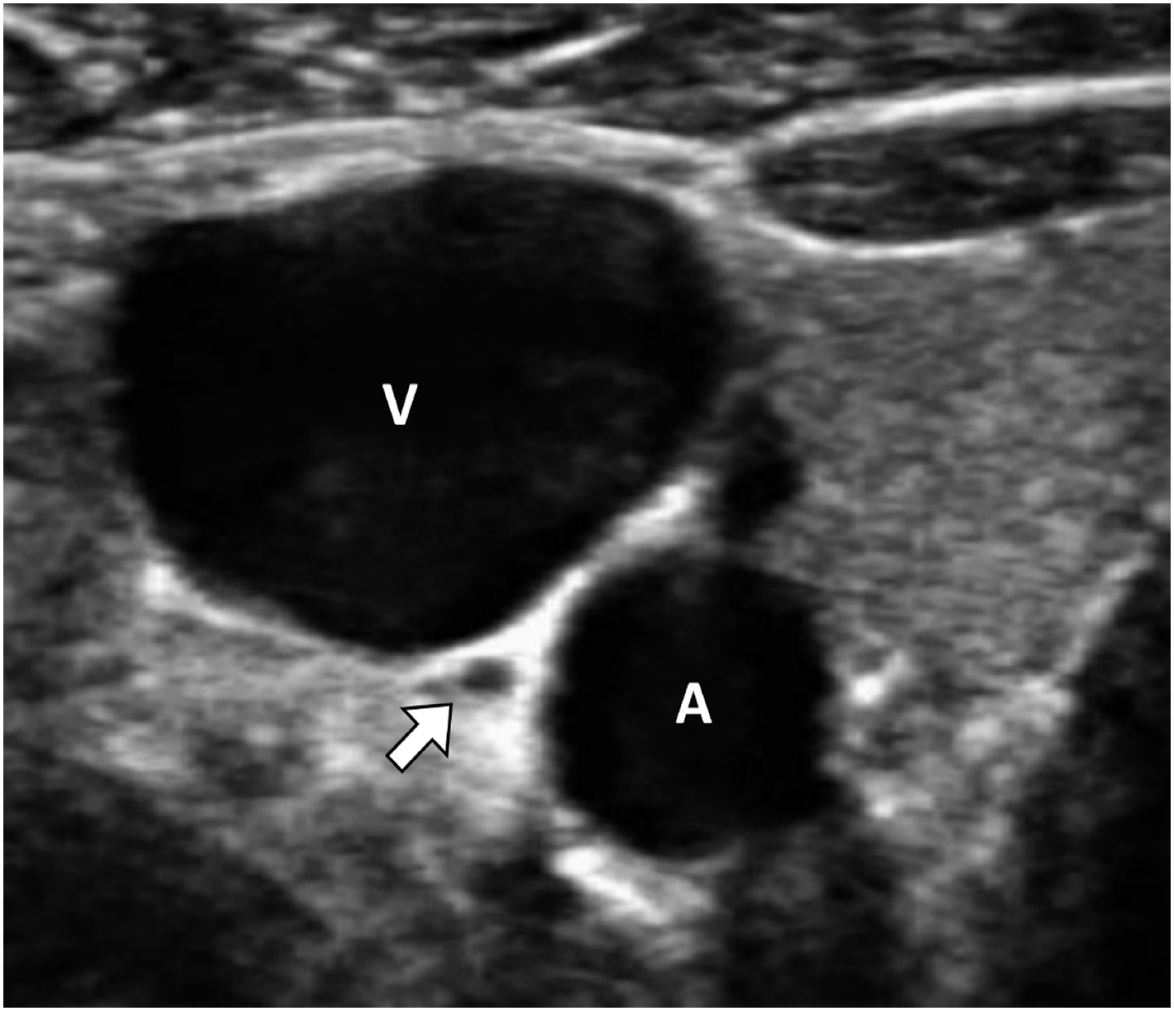
Transverse cross-sectional image of the vagus nerve by carotid ultrasound. The vagus nerve is shown as a small, rounded, hypoechoic structure between the carotid artery and jugular vein. The cross-sectional area of the vagus nerve was measured by manual tracing along the medial side of the hyperechoic epineural rim. The arrow indicates the vagus nerve. A, common carotid artery; V, jugular vein.

Carotid ultrasonography was performed using a Voluson E8 Expert system (GE Healthcare Japan, Tokyo, Japan) using a 9L-D linear probe (3–8 MHz). The examinations were conducted by one of two trained technologists. The participants were in the supine position with the neck slightly extended. Short-axis (transverse cross-sectional) images of the right and left vagus nerves at the thyroid level were obtained (Fig 1). Color doppler was used to differentiate between the nerve structures and the blood vessels. To minimize excessive transducer pressure and potential vagus nerve compression, the examiner ensured that the internal jugular vein remained visibly distended throughout the examination. Images were exported for offline analysis using EV-Insight software (PSP, Tokyo, Japan). A single experienced technologist, who was not the examiner and was blinded to the ECG results, measured the VN-CSA by manually tracing the inner edge of the hyperechoic epineural rim (measured in mm^2^ to one decimal place).

Measurement reliability was assessed using the intraclass correlation coefficient (ICC) and Bland–Altman analysis. The methods and results are shown in S2 File. Intra-rater and inter-rater reliability were approximately 0.6 for both right and left, which was judged to be “moderate” according to Koo & Li (2016) [25]. Bland–Altman analysis revealed fixed bias in some conditions, but no proportional bias was observed. The analysis was conducted under the assumption that the measured values were primarily affected by random measurement errors.

### ECG parameters

Twelve-lead ECG (FCP-8800, Fukuda Denshi, Tokyo, Japan) was recorded over 10 seconds with standard sensitivity (10 mm/mV) at 25 mm/s in the supine position. The heart rate, R–R interval, P–R interval, QRS duration, Bazett-corrected Q–T interval and Fridericia corrected Q–T interval were automatically derived. The Bazett and Fridericia-corrected J–T interval was calculated as (corrected Q–T interval – QRS duration). The maximum and minimum P-wave intervals and maximum P-wave amplitudes were also recorded, and leads without valid measurements were excluded. Atrial fibrillation (AF) was defined as AF/atrial flutter (Minnesota code: 8−3) based on the automated analysis. Note that the pre-measurement conditions and the ECG acquisition time were not standardized across participants.

### Other assessments

Height and weight were measured without shoes and in light clothing to calculate the body mass index (BMI). Hypertension was defined as a blood pressure of ≥140/90 mmHg and/or current treatment with antihypertensive agents. Resting blood pressure was measured three times using an automated device in the sitting position after 5 minutes of rest, and the average of the three measurements was used in the analyses. Diabetes mellitus was defined as a random blood glucose concentration of ≥200 mg/dL, hemoglobin A1c (HbA1c) of ≥6.5%, and/or current treatment with antidiabetic medication. The plasma glucose concentration and HbA1c were measured by an enzymatic method at a commercial laboratory (LSI Medience, Tokyo, Japan) within 24 hours after blood collection. Dyslipidemia was defined as a self-reported medical history and/or current treatment with antihyperlipidemic drugs. Current drinking was defined as drinking on a regular basis once a month. From the medication records, the use of cardiac antiarrhythmic drugs, Na^+^ channel blockers, β-blockers, K^+^ channel blockers, and non-dihydropyridine Ca^2+^ channel blockers, which are classified as Vaughan Williams classification I to IV [26], as well as cardiac glycosides and ivabradine, was defined as the “use of affects the cardiac action potential medication.” The classification and breakdown of affects the cardiac action potential medication is shown in S3 Table. Medical history, drinking habits, and smoking history were evaluated using patient-completed questionnaires, and medication status was determined by consulting the patients' medication records.

### Statistical analysis

The association between VN-CSA and ECG parameters was explored using two methods. Firstly, the right and left VN-CSA were divided into tertiles: right tertile (RT)1, RT2, and RT3, and left tertile (LT)1, LT2, and LT3. Baseline characteristics (e.g., age, sex, height, weight, BMI, blood pressure, HbA1c, comorbidities, and habits) are presented as the median [interquartile range (IQR)] or n (%). Comparisons were performed using the Kruskal–Wallis test for continuous variables and the chi-square test for categorical variables. Analysis of covariance (ANCOVA) was used to compare ECG parameters among the tertiles, adjusting for age, sex, height, weight, and use of affects the cardiac action potential medication. Partial eta-squared was calculated as an effect size to indicate the strength of the association between the VN-CSA tertiles and each ECG parameter. In multiple comparisons where a single parameter was repeated three times, Bonferroni correction was applied. As supplementary information, to examine the influence of sex on the results, we conducted a two-way analysis of variance (ANOVA) with the VN-CSA and sex as independent variables and ECG parameters as the dependent variable, examining the main effects of the VN-CSA and sex, as well as the interaction between the VN-CSA and sex.

Secondly, to examine the quantitative associations between variables, an analysis was conducted treating VN-CSA as a continuous variable. Linear regression analysis was used, with VN-CSA, age, sex, weight, and use of affects the cardiac action potential medication as explanatory variables, and each ECG parameter as the dependent variable. We created models with VN-CSA as the linear model and as the quadratic model. In the quadratic model, we used a central variable obtained by subtracting the average values for each sex from the VN-CSA. Height was excluded from the covariables because it was considered to have a strong correlation with weight. Assuming that the association between ECG parameters differs between the right and left sides, the main analysis was performed separately for the left and right sides. As supplementary information, an additional analysis was performed in which both sides were included as explanatory variables simultaneously in the linear model. The B coefficient [95% confidence interval (CI)] and p-value were calculated.

All statistical analyses were performed using IBM SPSS Statistics, version 27 (IBM Japan, Tokyo, Japan), with p < 0.05 considered statistically significant.

## Results

After excluding 3 participants with pacemaker implants, 14 with AF on ECG, and 2 without height/weight data, 183 participants were included in the final analysis.

The median [IQR] of the right VN-CSA was 1.00 [0.90–1.20] mm^2^ (mean ±standard deviation: 1.04 ± 0.24 mm^2^), and that of the left VN-CSA was 0.90 [0.80–1.10] mm^2^ (0.97 ± 0.22 mm^2^). The right and left the VN-CSA showed a weak correlation with a Pearson correlation coefficient of 0.309 (p < 0.001).

### VN-CSA tertiles and ECG parameters

**Fig 2:**
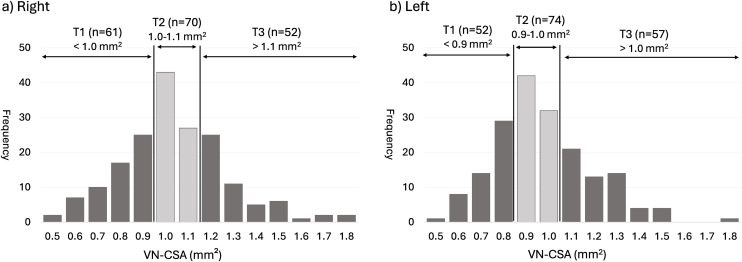
Distribution and tertile classification of the VN-CSA. The left and right VN-CSA were subjected to ranking and divided into three approximately equally sized groups: RT1, RT2, and RT3 on the right side and LT1, LT2, and LT3 of the left side. (a) right vagus nerve, (b) left vagus nerve. LT, left tertile; RT, right tertile; VN-CSA, vagus nerve cross-sectional area.

The VN-CSA distribution is shown in Fig 2. The right VN-CSA was classified into RT1 (<1.0 mm^2^, n = 61), RT2 (1.0–1.1 mm^2^, n = 70), and RT3 (>1.1 mm^2^, n = 52), and the left VN-CSA was classified into LT1 (<0.9 mm^2^, n = 52), LT2 (0.9–1.0 mm^2^, n = 74), and LT3 (>1.0 mm^2^, n = 57).

The characteristics of the participants by the VN-CSA tertiles are shown in Table 1. For the right VN-CSA, weight (p = 0.005) (T1 < T3, T2 < T3) and diastolic blood pressure (p = 0.026) (T1 > T3) differed significantly among the tertiles. Regarding the left VN-CSA, the proportion of males (p < 0.001) (T1 < T3), height (p = 0.016) (T1 < T3), weight (p = 0.023) (T1 < T3), and history of smoking (p = 0.003) (T1 < T3, T2 < T3) differed significantly among the tertiles.

**Table 1:** Characteristics of the study participants by VN-CSA tertiles.

	Overall	Right vagus nerve				Left vagus nerve			
		T1	T2	T3	p-value	T1	T2	T3	p-value
n	183	61	70	52		52	74	57	
Age (years), Median [IQR]	75.0[72.0–79.0]	75.0[71.0–78.0]	75.0[72.0–79.3]	75.0[73.0–79.8]	0.806	75.5[73.0–80.8]	75.0[72.0–79.3]	74.0[71.0–78.0]	0.191
Male sex, n (%)	82 (44.8)	23 (37.7)	29 (41.4)	30 (57.7)	0.080	13 (25.0)	33 (44.6)	36 (63.2)	<0.001
Height (cm), Median [IQR]	155.0[149.0–163.0]	155.0[149.0–161.0]	153.5[147.0–162.3]	158.0[151.0–164.8]	0.193	151.0[147.0–160.0]	156.0[150.0–164.0]	157.0[151.0–164.0]	0.016
Weight (kg), Median [IQR]	57.0[51.0–67.0]	55.0[49.5–65.5]	56.0[50.0–64.3]	64.5[55.0–70.0]	0.005	54.0[48.0–66.8]	57.0[51.8–65.3]	60.0[54.5–69.0]	0.023
BMI (kg/m2), Median [IQR]	23.7[21.4–26.2]	22.7[20.5–26.1]	23.6[21.4–25.6]	24.3[22.1–27.2]	0.052	23.6[21.0–25.7]	23.6[21.0–25.8]	23.8[22.1–26.7]	0.270
Systolic blood pressure (mmHg), Median [IQR]	148.3[136.0–162.0]	148.3[135.3–162.5]	150.2[137.8–163.2]	146.2[133.8–158.8]	0.397	151.2[138.1–165.1]	148.7[133.6–161.3]	147.7[136.8–159.2]	0.360
Diastolic blood pressure (mmHg), Median [IQR]	75.0[68.7–82.7]	77.3[71.5–86.7]	76.0[70.6–82.4]	71.0[64.1–80.6]	0.026	74.0[67.3–84.6]	75.3[69.6–82.4]	76.0[69.0–82.3]	0.997
Hypertension, n (%)	145 (79.2)	44 (72.1)	58 (82.9)	43 (82.7)	0.246	43 (82.7)	54 (73.0)	48 (84.2)	0.223
HbA1c (%), Median [IQR]	5.60[5.30–6.10]	5.50[5.20–6.10]	5.60[5.30–5.93]	5.60[5.40–6.10]	0.412	5.60[5.30–5.90]	5.60[5.30–6.13]	5.60[5.30–6.10]	0.817
Diabetes mellitus, n (%)	30 (16.4)	9 (14.8)	12 (17.1)	9 (17.3)	0.914	6 (11.5)	14 (18.9)	10 (17.5)	0.524
Dyslipidemia, n (%)	71 (38.8)	22 (36.1)	25 (35.7)	24 (46.2)	0.437	22 (42.3)	30 (40.5)	19 (33.3)	0.582
Current drinking, n (%)	89 (48.6)	26 (42.6)	34 (48.6)	29 (55.8)	0.379	19 (36.5)	42 (56.8)	28 (49.1)	0.082
History of smoking, n (%)	72 (39.3)	19 (31.1)	26 (37.1)	27 (51.9)	0.070	11 (21.2)	31 (41.9)	30 (52.6)	0.003
Affects the cardiac action potential medications, single or combined use, n (%)	16 (8.7)	5 (8.2)	8 (11.4)	3 (5.8)	0.540	7 (13.5)	3 (4.1)	6 (10.5)	0.156
Na^+^ channel blockers, n (%)	5 (2.7)	2 (3.3)	1 (1.4)	2 (3.8)	–	1 (1.9)	0 (0.0)	4 (7.0)	–
β-blockers, n (%)	13 (7.1)	3 (4.9)	7 (10.0)	3 (5.8)	–	5 (9.6)	2 (2.7)	6 (10.5)	–
Ca^2 +^ channel blockers, n (%)	3 (1.6)	1 (1.6)	2 (2.9)	0 (0.0)	–	1 (1.9)	1 (1.4)	1 (1.8)	–

VN-CSA, vagus nerve cross-sectional area; IQR, interquartile range; BMI, body mass index; HbA1c, hemoglobin A1c.

**Fig 3:**
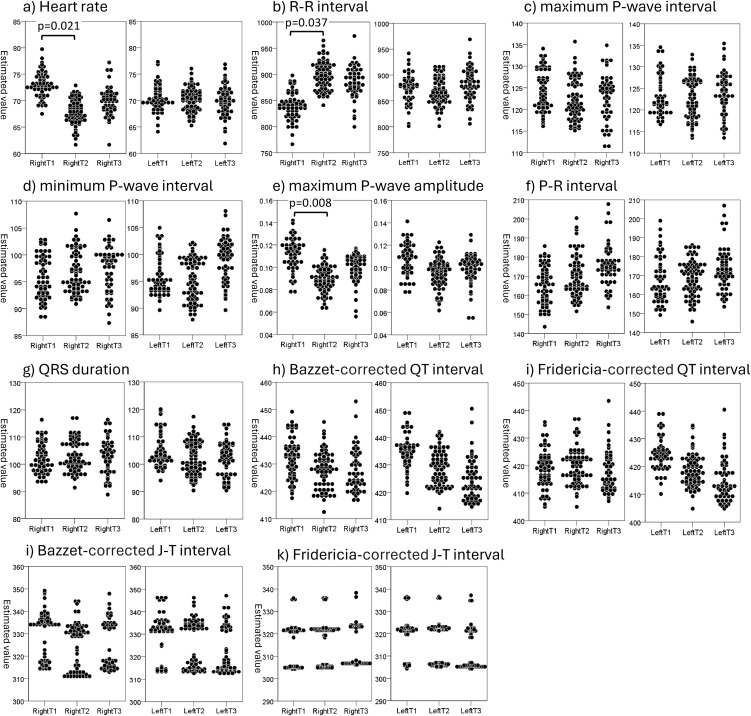
Multiple comparison of the estimated ECG parameter values by VN-CSA tertiles. Multiple comparisons among three groups (T1 vs T2, T1 vs T3, T2 vs T3) were evaluated using the Bonferroni method. (a) heart rate, (b) R–R interval, (c) maximum P-wave interval, (d) minimum P-wave interval, (e) maximum P-wave amplitude, (f) P–R interval, (g) QRS duration, (h) Bazett-corrected Q–T interval, (i) Fridericia -corrected Q–T interval, (j) Bazett-corrected J–T interval, (k) Fridericia -corrected J–T interval. ECG, electrocardiogram; RT, right tertile; VN-CSA, vagus nerve cross-sectional area.

The ANCOVA results of the association between the VN-CSA tertiles and ECG parameters with adjustment for age, sex, height, weight, and use of affects the cardiac action potential medication are shown in Table 2. Among the right VN-CSA tertiles, heart rate (p = 0.026), R–R interval (p = 0.040), and maximum P-wave amplitude (p = 0.009) significantly differed. The multiple comparison of the estimated ECG parameters by VN-CSA tertiles is shown in Fig 3. For each parameter, we performed three comparisons for each side and applied Bonferroni correction. As a result, we conducted a total of 66 comparisons. The RT1 group had a higher heart rate (p = 0.021), a shorter R–R interval (p = 0.037), and a higher maximum P-wave amplitude (p = 0.008) than the RT2 group. ECG parameters were not significantly different between RT1 and RT3. There were no significant associations among the left VN-CSA tertiles.

**Table 2:** ANCOVA results for the VN-CSA and ECG parameters.

	Right vagus nerve					Left vagus nerve				
	T1	T2	T3	p-value	Partial η²	T1	T2	T3	p-value	Partial η²
Heart rate (bpm), Mean [95%CI]	72.8 [70.2, 75.5]	67.9 [65.5, 70.4]	70.0 [67.1, 72.9]	0.026	0.041	69.8 [66.8, 72.7]	70.0 [67.6, 72.5]	70.7 [67.8, 73.5]	0.911	0.001
R–R interval (ms), Mean [95%CI]	841.6 [808.8, 874.3]	898.8 [868.2, 929.4]	881.1 [844.9, 917.2]	0.040	0.036	882.5 [845.7, 919.3]	869.9 [839.5, 900.2]	873.8 [838.3, 909.2]	0.872	0.002
maximum P-wave interval (ms), Mean [95%CI]	125.2 [121.7, 128.8]	123.2 [119.9, 126.5]	120.9 [116.9, 124.8]	0.276	0.015	124.9 [121.0, 128.9]	122.7 [119.4, 125.9]	122.3 [118.5, 126.1]	0.599	0.006
minimum P-wave interval (ms), Mean [95%CI]	96.6 [93.2, 99.9]	97.6 [94.4, 100.7]	95.6 [91.9, 99.3]	0.734	0.004	97.5 [93.8, 101.2]	95.2 [92.2, 98.3]	97.8 [94.2, 101.4]	0.489	0.008
maximum P-wave amplitude (mV), Mean [95%CI]	0.110 [0.100, 0.120]	0.089 [0.079, 0.098]	0.103 [0.092, 0.114]	0.009	0.053	0.109 [0.098, 0.120]	0.093 [0.084, 0.102]	0.100 [0.090, 0.111]	0.096	0.026
P–R interval (ms), Mean [95%CI]	167.1 [161.0, 173.3]	170.9 [165.1, 176.7]	172.2 [165.3, 179.0]	0.524	0.007	169.1 [162.2, 176.0]	170.1 [164.5, 175.8]	170.6 [164.0, 177.2]	0.950	0.001
QRS duration (ms), Mean [95%CI]	103.1 [98.7, 107.4]	104.2 [100.2, 108.2]	100.7 [96.0, 105.5]	0.553	0.007	106.8 [102.1, 111.5]	102.0 [98.2, 105.9]	100.3 [95.7, 104.8]	0.142	0.022
Bazett-corrected Q–T interval (ms), Mean [95%CI]	431.6 [425.8, 437.4]	428.2 [422.8, 433.6]	427.9 [421.5, 434.3]	0.617	0.006	432.6 [426.3, 439.0]	428.9 [423.6, 434.2]	426.5 [420.4, 432.7]	0.408	0.010
Fridericia-corrected Q–T interval (ms), Mean [95%CI]	418.5 [413.4, 423.5]	419.9 [415.2, 424.7]	417.9 [412.3, 423.4]	0.841	0.002	422.7 [417.2, 428.2]	418.4 [413.9, 423.0]	415.9 [410.6, 421.3]	0.233	0.017
Bazett-corrected J–T interval (ms), Mean [95%CI]	328.5 [322.8, 334.3]	324.0 [318.6, 329.4]	327.1 [320.7, 333.5]	0.502	0.008	325.9 [319.5, 332.3]	326.8 [321.6, 332.1]	326.3 [320.1, 332.4]	0.972	<0.001
Fridericia-corrected J–T interval (ms), Mean [95%CI]	315.4 [310.5, 320.4]	315.7 [311.1, 320.4]	317.1 [311.6, 322.6]	0.897	0.001	315.9 [310.4, 321.4]	316.4 [311.8, 320.9]	315.7 [310.4, 321.0]	0.979	<0.001

ANCOVA, analysis of covariance; VN-CSA, vagus nerve cross-sectional area; ECG, electrocardiogram; CI, confidence interval.

The results of a two-way ANOVA examining with the VN-CSA and sex as independent variables and ECG parameters as the dependent variable are shown in S4-1 and S4-2 Table. No significant association was observed among the right VN-CSA, heart rate, R–R interval, and maximum P-wave amplitude in either the main effects of sex or the interaction between the VN-CSA and sex.

### VN-CSA as continuous variables and ECG parameters

The results of the linear regression analyses, with VN-CSA, age, sex, weight, and use of affects the cardiac action potential medication as explanatory variables and ECG parameters as response variables, are shown in Table 3 (left and right separately). In the linear model, significant associations were observed between the left VN-CSA and QRS duration (p = 0.038). There was no significant association between the right VN-CSA and ECG parameters. The results of including both the right and left sides in the linear regression model simultaneously are shown in S5 Table. The results were similar to those obtained by analyzing the right and left sides separately. In the quadratic model, significant associations were observed between the right VN-CSA and Bazett-corrected Q–T interval (p = 0.015) and Fridericia-corrected Q–T interval (p = 0.048). There was no significant association between the left VN-CSA and ECG parameters.

**Table 3:** Linear regression analysis results for the VN-CSA and ECG parameters.

	Right VN-CSA			Left VN-CSA		
	B	[95%CI]	p-value	B	[95%CI]	p-value
**Linear model**						
Heart rate	−3.1	[-9.8, 3.5]	0.349	3.4	[-4.1, 10.9]	0.370
R–R interval	52.3	[-30.2, 134.8]	0.212	−26.6	[-120.3, 67.1]	0.576
maximum P-wave interval	−7.0	[-15.9, 1.8]	0.118	−9.1	[-19.1, 0.9]	0.075
minimum P-wave interval	−0.5	[-8.8, 7.9]	0.910	−3.3	[-12.7, 6.1]	0.489
maximum P-wave amplitude	−0.011	[-0.037, 0.014]	0.390	−0.016	[-0.045, 0.013]	0.265
P–R interval	11.5	[-3.9, 26.8]	0.142	1.2	[-16.3, 18.6]	0.894
QRS duration	1.0	[-9.8, 11.8]	0.853	−12.8	[-24.9, -0.7]	0.038
Bazett-corrected Q–T interval	0.6	[-13.9, 15.0]	0.938	−6.9	[-23.3, 9.4]	0.405
Fridericia-corrected Q–T interval	4.1	[-8.5, 16.8]	0.519	−9.6	[-23.8, 4.7]	0.186
Bazett-corrected J–T interval	−0.4	[-14.8, 13.9]	0.951	5.9	[-10.4, 22.2]	0.476
Fridericia-corrected J–T interval	3.1	[-9.3, 15.5]	0.620	3.2	[-10.8, 17.2]	0.650
**Quadratic model**						
Heart rate	13.1	[-4.1, 30.3]	0.134	18.6	[-6.3, 43.5]	0.142
R–R interval	−144.1	[-359.3, 71.1]	0.188	−151.3	[-464.4, 161.8]	0.342
maximum P-wave interval	−9.7	[-32.8, 13.5]	0.411	−15.9	[-49.3, 17.6]	0.350
minimum P-wave interval	−7.5	[-29.4, 14.4]	0.499	−11.1	[-42.7, 20.4]	0.488
maximum P-wave amplitude	0.064	[-0.003, 0.130]	0.060	0.085	[-0.011, 0.181]	0.084
P–R interval	8.6	[-31.6, 48.7]	0.675	−6.7	[-65.2, 51.8]	0.820
QRS duration	15.3	[-13.0, 43.6]	0.286	23.5	[-16.9, 63.9]	0.253
Bazett-corrected Q–T interval	46.4	[9.1, 83.7]	0.015	43.2	[-11.1, 97.6]	0.118
Fridericia-corrected Q–T interval	33.0	[0.2, 65.7]	0.048	26.9	[-20.6, 74.4]	0.265
Bazett-corrected J–T interval	31.1	[-6.4, 68.5]	0.103	19.7	[-34.7, 74.1]	0.475
Fridericia-corrected J–T interval	17.6	[-14.7, 50.0]	0.283	3.6	[-13.9, 21.1]	0.689

VN-CSA, vagus nerve cross-sectional area; ECG, electrocardiogram; CI, confidence interval.

## Discussion

In this study of 183 community-dwelling older adults, participants with a smaller right VN-CSA exhibited a higher heart rate, a shorter R–R interval, and a higher maximum P-wave amplitude than those with an intermediate VN-CSA in the tertile. However, linear regression analysis revealed no significant association between the right VN-CSA and heart rate, R–R interval, or maximum P-wave amplitude in either the linear or quadratic model. Conversely, the association between the left VN-CSA and QRS duration, which was not significant in the tertile analysis, was observed in the linear regression analysis.

### Factors associated with the VN-CSA

Previous studies evaluating the VN-CSA in relation to age, sex, height, weight, or BMI have produced inconsistent results. In general, the number of peripheral nerve axons and nerve cells decreases with age. One study reported a negative correlation between age and VN-CSA [22], while a meta-analysis found no association between the two [27]. In the present study, tertile grouping likewise revealed no significant differences in VN-CSA by age. Most studies have reported no association between weight, and BMI and VN-CSA [19,28,29], except a large-scale study involving 330 subjects [20]. However, in the present study, participants in the large-tertile groups tended to be taller, heavier, and more often male (for the left VN-CSA), suggesting that anthropometric indices may influence the VN-CSA.

There were some association between VN-CSA and factors other than ECG parameters including diastolic blood pressure and smoking history. Although there was a possibility of Type I errors due to multiple comparisons, heart rate might be a confounding factor in the association between the right VN-CSA and diastolic blood pressure. Additionally, a higher proportion of male might be a confounding factor in the association between the left VN-CSA and smoking history left CSA and smoking history.

The mean VN-CSA in the present study (1.04 mm^2^ on the right and 0.97 mm^2^ on the left) was smaller than that reported in some previous reports. Meta-analyses that the average VN-CSA area is approximately 2.0–2.5 mm² [2 7,30]. Reports from Japan and China tend to show smaller values compared to reports from Europe and the United States [15,21,31]. However, some reports suggested that only regional or racial differences could not account for variations in VN-CSA [10,32]. In this study, VN-CSA images were acquired at the thyroid level using a low-frequency probe, and offline measurements were performed. The reduced resolution resulting from the use of a low-frequency probe leads to blurring of the boundary between the epineural sheath and the surrounding tissue. Furthermore, the flattening of the vagus nerve caused by probe pressure alters the angle of incidence of ultrasound waves on the nerve's outer membrane, leading to a decrease in brightness due to anisotropy. The results of the ICCs were moderate, suggesting that the measurements in this study were likely primarily influenced by random error. Furthermore, fixed errors were observed in the Bland–Altman analysis. These findings regarding measurement reliability suggest that the possibility of underestimation of the VN-CSA due to the use of low-frequency probes or probe compression cannot be ruled out.

### Association between the VN-CSA and ECG parameters

Analysis by tertiles revealed a significant association between the right VN-CSA and heart rate and R–R interval, but this association was not significant in the linear regression analysis. While it cannot be ruled out that local differences may have been amplified by the intergroup comparison or that measurement errors may have influenced the regression analysis, this suggests that it is not simply nonlinear, but rather that a threshold-like may exist. One study using the head-up tilt test showed a negative correlation between right VN-CSA and heart rate in patients with Parkinson's disease [12]. Notably, a study using immunohistochemical and histochemical analyses revealed that the sinus node exhibited the highest density of neurons and a predominance of acetylcholinesterase-positive nerves, which are thought to be markers of parasympathetic function [33,34]. These findings suggest that the sinus node is particularly susceptible to changes in parasympathetic tone and could explain why a smaller right VN-CSA is associated with increased heart rate due to altered neural transmission from the vagus nerve.

Although there were no significant associations between VN-CSA and ECG parameters indicating ventricular activity (e.g., QRS duration, Q–T interval, J–T interval) in the ANCOVA. However, despite the influence of measurement errors, linear regression analysis revealed a significant association between the left VN-CSA and QRS duration in the linear model, suggesting that the true relationship between the left VN-CSA and QRS duration may have disappeared due to the grouping of continuous variables in the tertile analysis. It was reported that there was negative correlation between the left VN-CSA and HRV [24]. It is known that the right vagus nerve innervates the sinus node, while the left vagus nerve influences conduction at the atrioventricular node and ventricular contractility [5–9,35–37]. Furthermore, while heart rate is controlled by the sinus node, HRV reflects the transmission of electrical signals throughout the entire heart and is known to be influenced by structural changes in the heart [38,39]. The asymmetric connections of the right and left vagus nerve to the heart, as well as the differences in the properties of heart rate and HRV, may account for the observed differences in the associations between right VN-CSA and heart rate in this study and between left VN-CSA and HRV in previous reports [24]. The right VN-CSA may be associated with the electrical activity of the sinus node, while the left VN-CSA may be associated with the electrical activity of the ventricles. Nonetheless, because heart rate elevation is a known risk factor for all-cause mortality [40–43], clarifying whether a smaller VN-CSA predicts cardiovascular events in older adults would be an important next step. Moreover, further research in patients with underlying heart disease is needed to clarify the precise relationship between VN-CSA and ventricular function.

Linear regression analysis revealed a significant association between the right VN-CSA and corrected Q–T interval in the quadratic model, but these associations show sex differences and interaction with sex. The association between the right VN-CSA and corrected Q–T interval may be influenced by the ratio of male to female in the participants included in the analysis. Therefore, this study results prevent us from discussing the relationship between the VN-CSA and corrected Q–T interval.

Conversely, the left VN-CSA showed no significant association with P–R interval in this study. Generally, age-related fibrosis is the main cause of atrioventricular nodal conduction abnormalities in older adults. Only first-degree atrioventricular block was observed among the participants in the present study, which was considered to be a possible age-related ECG change. Future research involving patients with more advanced atrioventricular block may help to clarify whether morphological changes in the left vagus nerve also contribute to conduction abnormalities beyond structural aging.

### Association with P-waves

The group with the smallest right VN-CSA exhibited a higher maximum P-wave amplitude, which is often used as a surrogate for right atrial enlargement. While hypertension is a common contributor to atrial enlargement, there was no significant difference in blood pressure between the small- and intermediate-tertile groups. The P-wave amplitude observed in this study was within the normal range across all tertiles, suggesting that it is unlikely to directly reflect right atrial enlargement caused by simple pressure overload. Additionally, linear regression analysis revealed no significant association between the right VN-CSA and maximum P-wave amplitude in either the linear or quadratic model. These results were consistent with those for heart rate and R–R interval. This suggests that the association between the right VN-CSA and maximum P wave amplitude may involve a threshold. Acetylcholinesterase-positive neurons, which serve as markers of parasympathetic function, are abundant in the atria, second only to the conduction system, and are more numerous in the right atrium than in the left [44]. This suggests that, like the sinoatrial node, the atria (particularly the right atrium) are sensitive to changes in parasympathetic tone. The right VN-CSA may be associated with the electrical activity of the atria. Age-related structural changes in the atria have been reported to cause conduction disturbances, leading to an increase in areas of low potential and a decrease in conduction velocity [45,46]. We previously reported that the right VN-CSA was independently associated with AF in patients with ischemic stroke or transient ischemic attack [47]. These findings suggest that morphological and structural changes in the vagus nerve may alter vagal nerve transmission, and, when combined with age-related structural remodeling, lead to electrical heterogeneity within the atria. Furthermore, given that certain P-wave indices are known risk factors for AF [48–50], investigating whether the VN-CSA predicts AF or other supraventricular arrhythmias in older adults would be a valuable next step.

### In association with vagus nerve stimulation

In recent years, vagus nerve stimulation (VNS) has been used to treat a variety of conditions. Transcutaneous VNS acts primarily through afferent circuits to the brainstem; therefore, direct side effects on the heart are minimal, and it has been reported that stimulation of either the left or right side is highly tolerable and safe in humans [51,52]. On the other hand, left-sided stimulation has traditionally been preferred due to concerns about the cardiac effects of right-sided stimulation in invasive VNS. In peripheral neuropathy, there is an uneven decrease in nerve conduction velocity and changes in conduction time [53]. In invasive VNS, stimulation of vagus nerves that have undergone morphological and structural changes may not only fail to produce the anticipated effects but also lead to irregularities in stimulation intensity due to unstable signal transmission, potentially resulting in proarrhythmic effects.

### Limitations

This study has some limitations that should be considered. First, as aforementioned images of the vagus nerve were acquired using a low-frequency probe, and statistical analysis was performed based on offline measurements taken by a single evaluator. The results of the ICCs and Bland–Altman analyses indicate that it is not possible to eliminate the effects of random and fixed errors in the measurements caused by the reduced resolution of the low-frequency probe. Furthermore, the low reliability of the measurements may result in an underestimation of the true association in the linear regression analysis. Although the observed regression coefficients are theoretically attenuated, the 95% CI for the ICCs was wide in some conditions, making it difficult to accurately estimate the degree of attenuation. Second, although ECG measurements were largely automated, certain leads could not be measured automatically, potentially affecting accuracy. Additionally, ECG recording conditions are not standardized for all participants. Third, sex influenced both the VN-CSA and ECG parameters. Due to sample size limitations, we adopted a two-way ANOVA with added sex instead of the sex-stratified analysis, to examine the influence of sex on the results. The results of a two-way ANOVA, there was no significant association among the variables in either the main effects of sex or the interaction between the VN-CSA and sex in the ECG parameters that were significant in ANCOVA. Further investigation is needed to determine if the difference in ECG parameters resulting from cross-sectional area size is due to the parasympathetic biology or wholly driven by sex differences in nerve size. Fourth, because this study is a cross-sectional study, it cannot establish a causal relation between left and right VN-CSA and ECG parameters. Fifth, this study was conducted on community-dwelling older adults. The result of this study could not be applied to all age populations. To utilize VN-CSA measurement for routine clinical use, further research is needed involving healthy individuals (including younger adults) and patients with various diseases, addressing factors such as intra-individual variability and prognostic significance.

## Conclusion

In community-dwelling older adults, a smaller right VN-CSA was associated with a higher heart rate, shorter R–R interval, and higher P-wave amplitude than the intermediate VN-CSA in tertile methods. Furthermore, a significant linear association between the left VN-CSA and QRS duration was revealed by linear regression analysis. These findings suggest that the size of the vagus nerve may have different effects on cardiac electrical activity in the left and right sides. The right VN-CSA may be associated with the electrical activity of the sinus node and atria, while the left VN-CSA may be associated with the electrical activity of the ventricles.

## Supporting information

S1 Checklist STROBE Statement. (DOCX)

S2 File Measurement Reliability. (DOCX)

S3 Table Classification and breakdown of affects the cardiac action potential medication. (XLSX)

S4 Table S4.1 Table. Two-way analysis of variance for the right VN-CSA and sex. S4.2 Table. Two-way analysis of variance for the left VN-CSA and sex.
(XLSX)

S5 Table Linear regression analysis results for the VN-CSA and ECG parameters: Both left and right VN-CSA. (XLSX)
