# Supplementary material for: Impact of vagus nerve cross-sectional area on electrocardiogram parameters in community-dwelling older adults: The YAHABA study
Source: PLoS One. 2026 Jul 10;21(7):e0353473. doi: 10.1371/journal.pone.0353473 (PMC13354007; doi:10.1371/journal.pone.0353473)
Supplement: S1 Checklist — (DOCX) [file pone.0353473.s001.docx]

S1 Checklist. STROBE Statement—checklist of items that should be included in reports of observational studies

|  | Item No. | Recommendation | | Page  No. | Relevant text from manuscript |
| --- | --- | --- | --- | --- | --- |
| **Title and abstract** | 1 | (*a*) Indicate the study’s design with a commonly used term in the title or the abstract | | 1 | Full title: Impact of vagus nerve cross-sectional area on electrocardiogram parameters in community-dwelling older adults: The YAHABA study  Short title: Vagus nerve cross-sectional area and electrocardiogram parameters |
|  |  | (*b*) Provide in the abstract an informative and balanced summary of what was done and what was found | | 2,3 | See abstract. |
| Introduction | | |  | | |
| Background/rationale | 2 | Explain the scientific background and rationale for the investigation being reported | | 3,4 | The association between the vagus nerve and cardiac electrical activity, it has been reported vagal stimulation have indicated that vagus nerve stimulation reduces heart rate with right-sided stimulation and induces atrioventricular block with left-sided stimulation. However, the impact of vagus nerve cross-sectional area on cardiac electrical activity has not been investigated. |
| Objectives | 3 | State specific objectives, including any prespecified hypotheses | | 4 | Hypothesis: The cross-sectional area of the vagus nerve impacts the cardiac autonomic nervous system.  Objective: The aim of this study was to comprehensively investigate the association between the vagus nerve cross-sectional area evaluated by carotid ultrasonography and electrocardiogram parameters in community-dwelling older adults, thereby clarifying the influence of vagus nerve cross-sectional area on cardiac electrical activity. |
| Methods | | |  | | |
| Study design | 4 | Present key elements of study design early in the paper | | 2 | A cross-sectional study examining the association between vagus nerve cross-sectional area and 12-lead electrocardiogram in community-dwelling older adults. |
| Setting | 5 | Describe the setting, locations, and relevant dates, including periods of recruitment, exposure, follow-up, and data collection | | 5 | The study was conducted in Yahaba, Iwate, Japan, from January to March of 2023. |
| Participants | 6 | (*a*) *Cohort study*—Give the eligibility criteria, and the sources and methods of selection of participants. Describe methods of follow-up  *Case-control study*—Give the eligibility criteria, and the sources and methods of case ascertainment and control selection. Give the rationale for the choice of cases and controls  *Cross-sectional study*—Give the eligibility criteria, and the sources and methods of selection of participants | | 5 | The target population for this study included individuals aged ≥65 years living in Yahaba, Iwate, Japan. Of these, 962 participants were included in the baseline study of the YAHABA Study from 2016 to 2018, and 762 participants were included in the follow-up study from 2021 to 2023.  The present study enrolled participants from the 2021-2023 follow-up study who underwent carotid ultrasound and electrocardiogram examinations during the study conducted from January to March of 2023. Individuals with a history of pacemaker implantation, those with atrial fibrillation detected on electrocardiogram, and those with missing data in any variable required for analysis were excluded from the study. |
|  |  | (*b*) *Cohort study*—For matched studies, give matching criteria and number of exposed and unexposed  *Case-control study*—For matched studies, give matching criteria and the number of controls per case | | N/A | N/A |
| Variables | 7 | Clearly define all outcomes, exposures, predictors, potential confounders, and effect modifiers. Give diagnostic criteria, if applicable | | 5-9 | Exposure: Cross-sectional area of the left and right vagus nerve measured by ultrasonography.  Outcome: Electrocardiogram parameters: heart rate, R–R interval, P–R interval, QRS duration, and Bazett-corrected Q–T interval, Fridericia-corrected Q–T interval, Bazett-corrected J–T interval, Fridericia-corrected J–T interval, maximum and minimum P-wave durations and maximum P-wave amplitudes.  Potential confounder: Age, sex, height, weight, body mass index, hypertension, systolic blood pressure, diastolic blood pressure, diabetes mellitus, hemoglobin A1c, dyslipidemia, current drinking, history of smoking and using of affects the cardiac action potential medication. |
| Data sources/ measurement | 8* | For each variable of interest, give sources of data and details of methods of assessment (measurement). Describe comparability of assessment methods if there is more than one group | | 5-7 | Cross-sectional area of the vagus nerve: One of the two the technicians recorded images of the cross-sectional area of the vagus nerve at the thyroid level on each side during carotid ultrasonography and saved the images on the ultrasound devices. A different technician conducted offline measurements of all saved images using image analysis software, and the obtained values were used for analysis.  Electrocardiogram parameters: A standard 12-lead electrocardiogram was automatically recorded for 10 seconds. The heart rate, R–R interval, P–R interval, QRS duration, Bazett-corrected Q–T interval, and Fridericia-corrected Q–T interval used measurements that were automatically analysed. The Bazett- and Fridericia-corrected J–T interval was calculated as corrected QT minus QRS. The P wave width and amplitude were determined using the maximum and minimum durations and maximum amplitude across all leads, with the exception of those where measurements could not be obtained. Atrial fibrillation was defined as atrial fibrillation/atrial flutter (Minnesota Code 8-3) using the Fukuda Denshi Minnesota Code.  Potential confounder: Height and weight were measured to calculate the body mass index. Hypertension was defined as a blood pressure of ≥140/90 mmHg and/or current treatment with antihypertensive agents. Resting blood pressure was measured three times in the sitting position after 5 minutes of rest, and the average of the three measurements was used in the analyses. Diabetes mellitus was defined as a random blood glucose concentration of ≥200 mg/dL, hemoglobin A1c of ≥6.5%, and/or current treatment with antidiabetic medication. The plasma glucose concentration and HbA1c were measured by an enzymatic method after blood collection. Dyslipidemia was defined as a self-reported medical history and/or current treatment with antihyperlipidemic drugs. Current drinking was defined as drinking on a regular basis once a month. From the medication records, the use of cardiac antiarrhythmic drugs, Na+ channel blockers, β-blockers, K+ channel blockers, and non-dihydropyridine Ca2+ channel blockers, which are classified as Vaughan Williams classification I to IV, as well as cardiac glycosides and ivabradine, was defined as the "use of affects the cardiac action potential medication". Medical history, drinking habits, and smoking history were evaluated using patient-completed questionnaires, and medication status was determined by consulting the patients’ medication records. |
| Bias | 9 | Describe any efforts to address potential sources of bias | | 21 | The bias in the method of measuring vagus nerve cross-sectional area was mentioned in the limitations. |
| Study size | 10 | Explain how the study size was arrived at | | N/A | There are no reports examining the association between vagus nerve cross-sectional area and electrocardiogram measurements, and no statistical sample size analysis has been conducted. |

Continued on next page

| Quantitative variables | 11 | Explain how quantitative variables were handled in the analyses. If applicable, describe which groupings were chosen and why | 7-9 | Age, height, weight, body mass index, systolic blood pressure, diastolic blood pressure, hemoglobin A1c, and electrocardiogram measurements were analysed as continuous variables. |
| --- | --- | --- | --- | --- |
| Statistical methods | 12 | (*a*) Describe all statistical methods, including those used to control for confounding | 7-9 | We performed comparisons of characteristics among the tertiles of the cross-sectional area of the vagus nerve using the Kruskal–Wallis test for continuous variables and the chi-square test for categorical variables.  The analysis of covariance was used to compare electrocardiogram parameters among the tertiles, adjusting for age, sex, height, and weight. Multiple comparisons were evaluated using the Bonferroni method.  To examine the influence of sex on the results, we conducted a two-way analysis of variance, examined the main effects of the vagus nerve cross-sectional area and sex, as well as the interaction between the vagus nerve cross-sectional area and sex.  Additionally, linear regression analysis in the linear model and the quadratic model was used, with vagus nerve cross-sectional area (left and right separately), age, sex, weight, and use of affects the cardiac action potential medication as explanatory variables, and each electrocardiogram parameter as the dependent variable. An additional analysis was performed in which both sides were included as explanatory variables simultaneously in linear model.  All statistical analyses with p < 0.05 were considered statistically significant. |
|  |  | (*b*) Describe any methods used to examine subgroups and interactions | N/A | There have been few reports on the association between vagus nerve cross-sectional area and electrocardiogram measurements, and no investigation has been considered into effect-modifying factors. |
|  |  | (*c*) Explain how missing data were addressed | 6,7 | For P-wave measurements by electrocardiogram, we excluded leads for which automated measurements were not available, and used the maximum and minimum values among the leads for which automated measurements were available. In the event that any of the other variables were missing, all variables were excluded from the analysis. |
|  |  | (*d*) *Cohort study*—If applicable, explain how loss to follow-up was addressed  *Case-control study*—If applicable, explain how matching of cases and controls was addressed  *Cross-sectional study*—If applicable, describe analytical methods taking account of sampling strategy | N/A |  |
|  |  | (*e*) Describe any sensitivity analyses | N/A | This was not conducted as it was an exploratory study. |
| Results | | | | |
| Participants | 13* | (a) Report numbers of individuals at each stage of study—eg numbers potentially eligible, examined for eligibility, confirmed eligible, included in the study, completing follow-up, and analysed | 9 | Of the 202 subjects in this study, 183 were included in the final analysis after excluding 3 participants with pacemaker implants, 14 with atrial fibrillation on electrocardiogram, and two without height/weight data. |
|  |  | (b) Give reasons for non-participation at each stage | 9 | Excluding 3 participants with pacemaker implants, 14 with atrial fibrillation on electrocardiogram, and two without height/weight data. |
|  |  | (c) Consider use of a flow diagram | N/A | A flow diagram was not created because the exclusion criteria were simple. |
| Descriptive data | 14* | (a) Give characteristics of study participants (eg demographic, clinical, social) and information on exposures and potential confounders | 9-11  Table 1 | The characteristics of the participants were shown in Table 1. For the right vagus nerve cross-sectional area was associated weight and diastolic blood pressure, the left vagus nerve cross-sectional area was associated gender, height, weight, and history of smoking. These results suggest that sex, height, and weight may be confounding the data. |
|  |  | (b) Indicate number of participants with missing data for each variable of interest | N/A | No missing data |
|  |  | (c) *Cohort study*—Summarise follow-up time (eg, average and total amount) | N/A |  |
| Outcome data | 15* | *Cohort study*—Report numbers of outcome events or summary measures over time | N/A |  |
|  |  | *Case-control study—*Report numbers in each exposure category, or summary measures of exposure | N/A |  |
|  |  | *Cross-sectional study—*Report numbers of outcome events or summary measures | 11-13  Table 2  Figure 3 | The estimated mean of the electrocardiogram parameters are shown in Table 2. The distribution of the electrocardiogram parameters was shown in Figure 3. |
| Main results | 16 | (*a*) Give unadjusted estimates and, if applicable, confounder-adjusted estimates and their precision (eg, 95% confidence interval). Make clear which confounders were adjusted for and why they were included | 11-15  Table 2  Figure 3  Table3 | The adjusted results were shown in Table 2, and the results of multiple comparisons were shown in Figure 3.  In linear regression analysis, the adjusted results were shown in Table 3. |
|  |  | (*b*) Report category boundaries when continuous variables were categorized | N/A | No conducted. |
|  |  | (*c*) If relevant, consider translating estimates of relative risk into absolute risk for a meaningful time period | N/A | This was not conducted as it was an exploratory study. |

Continued on next page

| Other analyses | 17 | Report other analyses done—eg analyses of subgroups and interactions, and sensitivity analyses | N/A |  |
| --- | --- | --- | --- | --- |
| Discussion | | | | |
| Key results | 18 | Summarise key results with reference to study objectives | 15-16 | In this study of 183 community-dwelling older adults, participants with a smaller right vagus nerve cross-sectional area exhibited a higher heart rate, a shorter R–R interval, and a higher maximum P-wave amplitude than those with an intermediate vagus nerve cross-sectional area in the tertile. However, linear regression analysis revealed no significant association between the right vagus nerve cross-sectional area and heart rate, R–R interval, or maximum P-wave amplitude in either the linear or quadratic model. Conversely, the association between the left vagus nerve cross-sectional area and QRS duration, which was not significant in the tertile analysis, was observed in the linear regression analysis. |
| Limitations | 19 | Discuss limitations of the study, taking into account sources of potential bias or imprecision. Discuss both direction and magnitude of any potential bias | 21,22 | First, images of the vagus nerve were acquired using a low-frequency probe, and statistical analysis was performed based on offline measurements taken by a single evaluator. The results of the ICCs and Bland–Altman analyses indicate that it is not possible to eliminate the effects of random and fixed errors in the measurements caused by the reduced resolution of the low-frequency probe. Furthermore, the low reliability of the measurements may result in an underestimation of the true association in the linear regression analysis. Second, although electrocardiogram measurements were largely automated, certain leads could not be measured automatically, potentially affecting accuracy. Additionally, electrocardiogram recording conditions are not standardized for all participants. Third, sex influenced both the vagus nerve cross-sectional area and electrocardiogram parameters. Due to sample size limitations, we adopted a two-way analysis of variance with added sex instead of the sex-stratified analysis, to examine the influence of sex on the results. The results of a two-way analysis of variance, there was no significant association among the variables in either the main effects of sex or the interaction between the vagus nerve cross-sectional area and sex in the electrocardiogram parameters that were significant in analysis of covariance. Fourth, because this study is a cross-sectional study, it cannot establish a causal relation between left and right vagus nerve cross-sectional area and electrocardiogram parameters. Fifth, this study was conducted on community-dwelling older adults. The result of this study could not be applied to all age populations. |
| Interpretation | 20 | Give a cautious overall interpretation of results considering objectives, limitations, multiplicity of analyses, results from similar studies, and other relevant evidence | 22 | In tertile method, the right vagus nerve cross-sectional area was associated with heart rate and P-wave amplitude. In the linear regression analysis, there was a significant association between the left vagus nerve cross-sectional area and QRS duration. These findings suggest that the size of the vagus nerve may have different effects on cardiac electrical activity in the left and right sides. The right vagus nerve cross-sectional area may be associated with the electrical activity of the sinus node and atria, while the left vagus nerve cross-sectional area may be associated with the electrical activity of the ventricles. |
| Generalisability | 21 | Discuss the generalisability (external validity) of the study results | 21,22 | This study was exploratory, so its results will need to be checked for external validity verification, as stated in the Discussion section. |
| Other information | |  | | |
| Funding | 22 | Give the source of funding and the role of the funders for the present study and, if applicable, for the original study on which the present article is based | N/A | I filled out the submission form following the submission guidelines. |

*Give information separately for cases and controls in case-control studies and, if applicable, for exposed and unexposed groups in cohort and cross-sectional studies.

**Note:** An Explanation and Elaboration article discusses each checklist item and gives methodological background and published examples of transparent reporting. The STROBE checklist is best used in conjunction with this article (freely available on the Web sites of PLoS Medicine at http://www.plosmedicine.org/, Annals of Internal Medicine at http://www.annals.org/, and Epidemiology at http://www.epidem.com/). Information on the STROBE Initiative is available at www.strobe-statement.org.
