# Supplementary material for: Impact of vagus nerve cross-sectional area on electrocardiogram parameters in community-dwelling older adults: The YAHABA study
Source: PLoS One. 2026 Jul 10;21(7):e0353473. doi: 10.1371/journal.pone.0353473 (PMC13354007; doi:10.1371/journal.pone.0353473)
Supplement: S2 File — (DOCX) [file pone.0353473.s002.docx]

**S2 File. Measurement Reliability**

**Methods**

Firstly, 50 cases were randomly selected from the 183 cases included in this study. The technologist who performed the original measurements re-measured the data, and the intra-rater reliability was calculated using Intraclass correlation coefficient (ICC) (1,1). In addition, a second independent technologist, who was blinded to the study aims and the original measurements, performed offline image measurements for the same cases, and inter-rater reliability was calculated using ICC (2,1).

Secondly, Bland–Altman analysis was performed. Fixed bias was evaluated using a one-sample t-test, and proportional bias was evaluated using linear regression analysis between the differences and the means of paired measurements.

All statistical analyses were performed using IBM SPSS Statistics, version 27 (IBM Japan, Tokyo, Japan), with p < 0.05 considered statistically significant.

**Results**

For 50 randomly selected cases, the intra-rater and inter-rater reliability ICCs were calculated separately for the left and right sides. The intra-rater reliability ICC (1,1) [95% CI] was 0.687 [0.508, 0.809] for the right side and 0.631 [0.431, 0.772] for the left side. The inter-rater reliability ICC (2,1) [95% CI] was 0.683 [0.337, 0.840] for the right side and 0.599 [0.237, 0.788] for the left side. The intra-rater and inter-rater reliability were judged to classified as be “moderate” according to Koo & Li (2016).

The results of the Bland–Altman analysis are shown in Figure. Mean bias [95% limit of agreement] for intra-rater reliability was -0.10 [-0.43, 0.23] for the right side and -0.01 [-0.37, 0.34] for the left side. Mean bias [95% limit of agreement] for the inter-rater reliability was -0.12 [-0.46, 0.22] for the right side and -0.10 [-0.39, 0.19] for the left side. Fixed bias was observed on the right side for the intra-rater reliability (right: p < 0.001; left: p = 0.586) and on both sides for the inter-rater reliability (right: p < 0.001; left: p < 0.001). However, no proportional bias was observed for either intra-rater reliability (right: β = 0.024, p = 0.870; left: β = -0.226, p = 0.115) or inter-rater reliability (right: β = -0.086, p = 0.554; left: β = 0.032, p = 0.827).

**
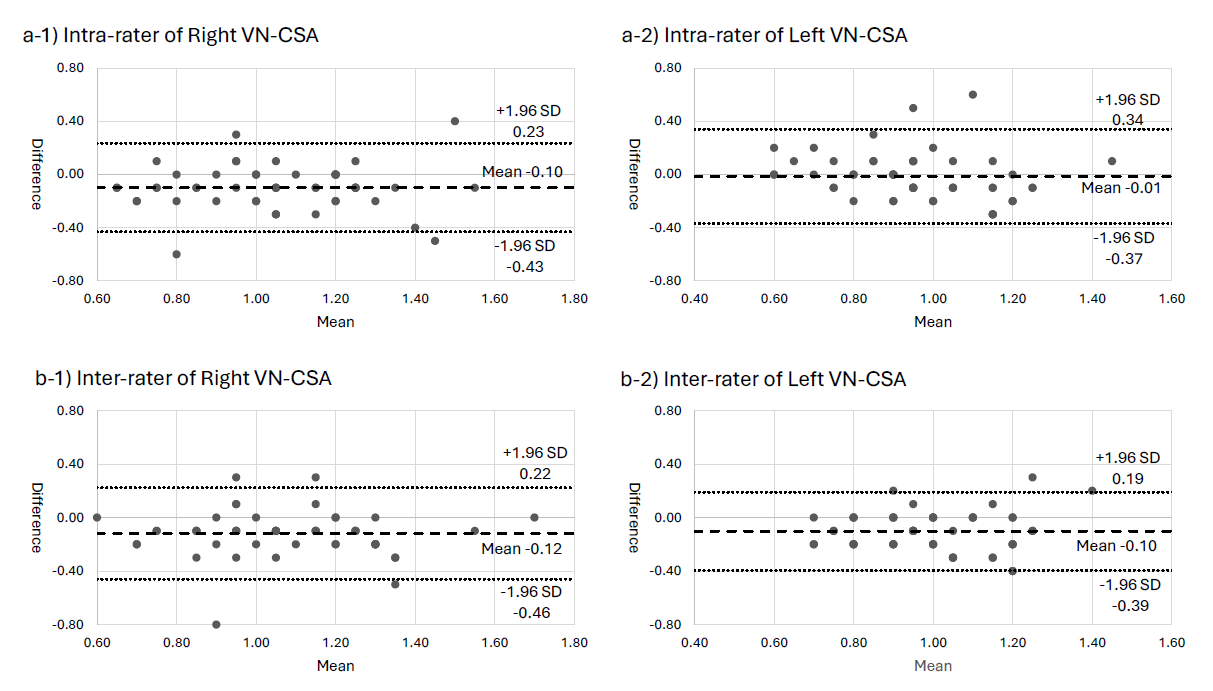
Figure. Bland–Altman plot.** Bland–Altman plots for intra-rater and inter-rater reliability. The dashed line represents the mean bias, and the dashed lines represent the 95% limits of agreement. (a-1) Intra-rater of Right VN-CSA, (a-2) Intra-rater of Left VN-CSA, (b-1) Inter-rater of Right VN-CSA, (b-2) Inter-rater of Left VN-CSA. VN-CSA, vagus nerve cross-sectional area; SD, standard deviation.
